# The Use of Borax and Boric Acid as Food Preservatives

**Published:** 1902-07

**Authors:** 


### The Use of Borax and Boric Acid as Food Preservatives.

Under the above title Vaughan & Veenboer, in *American Medicine* of March 15th, give an exhaustive review of experiments conducted by Liebrich and other observers, all of which bear out Liebrich's conclusions that boric acid is practically without effect upon the mucous membrane of the stomach and intestines, while the comparatively mild effects produced by borax upon those viscera are due to its alkalinity.

The action of the various digestive ferments is unaffected by boric acid in 5 per cent. solution or by borax in less than 0.25 per cent. solution, while borax in 0.5 per cent. solution interferes with both salivary and gastric digestion, though only to the degree that would be expected from its alkalinity. Experiments by Chittenden and Gies lead them to state, that "moderate doses of borax up to five grams per day, even when continued for some time, are without influence upon proteid metabolism."

Continuing, the authors give a detailed account of their own experiments, conducted to show the action and effect of these substances when used as preservatives, and they conclude that the use of boric acid or borax in the proportions specified by the English commission is clearly justified for the preservation of butter and cream, while they also sanction the use of 1.5 per cent. of these substances as a dusting powder for the surface of meats prior to long shipments. C. R. M.

